# Supplementary material for: Determinants of feto-maternal outcomes of antepartum hemorrhage among women who gave birth in Awi zone public hospitals, Ethiopia. A case-control study
Source: PLoS One. 2024 Jul 8;19(7):e0297700. doi: 10.1371/journal.pone.0297700 (PMC11230564; doi:10.1371/journal.pone.0297700)
Supplement: Attachment — Submitted filename: PLOS ONE Review.docx [file pone.0297700.s003.docx]

**PLOS ONE REVIEW REVIEWERS REPORT**

**General comments**

The authors put together a good manuscript on the determinants of feto-maternal outcomes of antepartum hemorrhage among women who gave birth in Awi Zone public hospitals , Ethiopia using a case-control methodology. The authors determined that the following were the significant determinants of APH; rural residence, Delay to seek care >12 hours, not having Antenatal care follow up and advanced maternal age. However they do not provide which factors weigh more heavily than others. This also would reflect on strategies to inform policy and practice. I suggest that from their analysis the authors identify the factors in terms of statistical weight and explain the variation and reasons why some factors weigh in heavily than others

**Specific comments**

In the paragraph on clinical related characteristics of the cases and the controls the authors state the following- “…With regard to parity 117(52.2%) of cases were grand multipara who gave birth to five or more baby followed by multi parity 91(40.6%) and primi-para 16 (7.2%) respectively…” The authors should revise the sentence to change from the use of the word multi parity to multiparous and primiparous women for clarity of the sentence.

The following paragraph also has a list of abbreviations D& C, HDp and C/D that need to be written in full for the first time to aid reviewers and readers follow and understand the article

In section 3.3 – The authors write that –“…Most of the women were attained their antenatal care follow up…”. Kindly review the sentence for clarity

In table 3 on Health system related factors of the cases and controls in Awi Zone Public Hospitals the authors have an abbreviation that is not clarified- LNMP. Kindly provide full details to allow an understanding of the abbreviation.
