# Supplementary material for: Determinants of feto-maternal outcomes of antepartum hemorrhage among women who gave birth in Awi zone public hospitals, Ethiopia. A case-control study
Source: PLoS One. 2024 Jul 8;19(7):e0297700. doi: 10.1371/journal.pone.0297700 (PMC11230564; doi:10.1371/journal.pone.0297700)
Supplement: Attachment — Submitted filename: Rebuttable letter.docx [file pone.0297700.s004.docx]

**Author response**

**Manuscript ID:** PONE-D-22-15417

**Manuscript title:** Determinants of Feto-Maternal Outcomes of Antepartum Hemorrhage Among Women Who Gave Birth in Awi Zone Public Hospitals, Ethiopia, 2021.

**Dear Editor,**

We thank you and the reviewers for your comments, which improved our manuscript. Please find below a point-by-point response to reviewer comments. The changes made to our manuscript within the document were highlighted in yellow.

Thank you!

Ambaye Minayehu (MSc)

On the behalf of all authors

**Editor(s)' Comments to Author:**

1. Please ensure that your manuscript meets PLOS ONE's style requirements, including those for file naming.

**Response:** thank you for the remark. The manuscript was reviewed and edited according to PLOS ONE’s style.

2. Thank you for stating the following in the Acknowledgments Section of your manuscript:

“We would like to thank Assosa University for financial support to this study and Debre Markos University for granting permission to this study. This study was funded by the Assosa University Research and Community Service Directorate”

We note that you have provided additional information within the Acknowledgements Section that is not currently declared in your Funding Statement. Please note that funding information should not appear in the Acknowledgments section or other areas of your manuscript. We will only publish funding information present in the Funding Statement section of the online submission form.

Please remove any funding-related text from the manuscript and let us know how you would like to update your Funding Statement. Currently, your Funding Statement reads as follows:

**Response:** thank you for the remark. As per recommendation, we removed funding-related texts from the manuscript. Here we would like to describe we received a small grant from Assosa University and Debre Markos University for data collection.

“NO - Include this sentence at the end of your statement: The funders had no role in study design, data collection, and analysis, decision to publish, or preparation of the manuscript.”

**Response:** thank you for the remark. Yes of course, even though we received a small grant from Assosa University and Debre Markos University for data collection, these institutions have no role in study design, data collection, and analysis, decision to publish, or preparation of the manuscript.”

Please include your amended statements within your cover letter; we will change the online submission form on your behalf.

**Response:** thank you for the remark. As per recommendation, we had put amended statements within the cover letter.

3. We note that you have indicated that data from this study are available upon request. PLOS only allows data to be available upon request if there are legal or ethical restrictions on sharing data publicly.

In your revised cover letter, please address the following prompts:

a) If there are ethical or legal restrictions on sharing a de-identified data set, please explain them in detail (e.g., data contain potentially sensitive information, data are owned by a third-party organization, etc.) and who has imposed them (e.g., an ethics committee). Please also provide contact information for a data access committee, ethics committee, or other institutional body to which data requests may be sent.

**Response:** thank you for the remark. Here, we would like to mention that anybody who wants the data set can get it from the corresponding author through an email address, otherwise there are no ethical and legal restrictions. Now as PLOS standard we had uploaded the minimal anonymized data set.

b) If there are no restrictions, please upload the minimal anonymized data set necessary to replicate your study findings as either Supporting Information files or to a stable, public repository and provide us with the relevant URLs, DOIs, or accession numbers. We will update your Data Availability statement on your behalf to reflect the information you provide.

**Response:** thank you for the remark. As per recommendation, we uploaded the minimal anonymized data set.

4. Please upload a new copy of Figure 1 as the detail is not clear. Please follow the link for more information:

**Response:** thank you for the remark. As per recommendation, now we upload a copy of figure 1 containing clear details.

5. **Review Comments to the Author**

**Reviewer #1: General comments**

The authors put together a good manuscript on the determinants of feto-maternal outcomes of antepartum hemorrhage among women who gave birth in Awi Zone public hospitals, Ethiopia using a case-control methodology. The authors determined that the following were the significant determinants of APH; rural residence, Delay to seek care >12 hours, not having Antenatal care follow-up, and advanced maternal age. However, they do not provide which factors weigh more heavily than others. This also would reflect on strategies to inform policy and practice. I suggest that from their analysis the authors identify the factors in terms of statistical weight and explain the variation and reasons why some factors weigh in more heavily than others.

**Response:** thank you for the remark. As per recommendation, we put by statistical weight by using the Adjusted odds ratio and confidence interval.

Advanced maternal age [AOR: 3.43, 95% CI 1.784- 6.59], Delay to seek care >12 hours [AOR: 2.57, 95% CI: 1.57- 4.23], No antenatal care follow-up [AOR: 2.5, 95% CI 1.49-4.2], and Rural residence [AOR: 1.706, 95%CI 1.09-2.66].

**Specific comments**

In the paragraph on clinical related characteristics of the cases and the controls, the authors state the following- “…With regard to parity 117(52.2%) of cases were grand multipara who gave birth to five or more baby followed by multi parity 91(40.6%) and primipara 16 (7.2%) respectively…” The authors should revise the sentence to change from the use of the word multi parity to multiparous and primiparous women for clarity of the sentence. The following paragraph also has a list of abbreviations D& C, HDP and C/D that need to be written in full for the first time to aid reviewers and readers follow and understand the article In section 3.3 – The authors write that –“…Most of the women were attained their antenatal care follow up…”. Kindly review the sentence for clarity

**Response:** thank you for the remark. As per recommendation, we change the words “multi parity and primiparity” to recommended words. Now we have introduced the abbreviations “D& C”, “C/D” and “HDP” first as dilatation and curettage (D&C), a cesarean delivery (C/D), and hypertensive disorder of pregnancy (HDP) respectively. We have also revised the sentence “…Most of the women were attained their antenatal care follow up…” per recommendation.
