# Supplementary material for: Determinants of feto-maternal outcomes of antepartum hemorrhage among women who gave birth in Awi zone public hospitals, Ethiopia. A case-control study
Source: PLoS One. 2024 Jul 8;19(7):e0297700. doi: 10.1371/journal.pone.0297700 (PMC11230564; doi:10.1371/journal.pone.0297700)
Supplement: Attachment — Submitted filename: Rebuttable letter.docx [file pone.0297700.s005.docx]

**Author response**

**Manuscript ID:**  [PONE-D-22-15417R1] - [EMID:8d5c1140a6335dc7]

**Manuscript title:** Determinants of feto-maternal outcomes of antepartum hemorrhage among women who gave birth in Awi zone public hospitals, Ethiopia. A case-control study.

**Dear Editor,**

We thank you and the reviewers for your comments, which improved our manuscript. Please find below a point-by-point response to the reviewers’ comments. The changes made to our manuscript within the document were highlighted in yellow.

Thank you,

Ambaye Minayehu (MSc)

On behalf of all authors.

**Authors’ response for Editor(s)' Comments**

- use the current report on maternal mortality (Trends in maternal mortality)

Thank you for the remark. Trends in are used maternal mortality as indicated.

-Include the regional (SSA) perspective. What are the current estimates and the changes overtime and the major causes?

Thank you for the remark. The regional perspective and changes over time, as well as its causes, had described.

- relatedly, include some statistics on Ethiopia - current estimates, causes, and policies in place (see also Reviewer 3 comment).

Thank you for the remark. We incorporate statistical estimates in Ethiopia.

- Describe your study variables and their measurements in this study including how they were initially collected and changed during analysis. It would be better to have them in a table.

Thank you for the remark. As indicated, we describe variables, their measurements, and the analysis method in the variable list and operational definition.

-In your conclusion (abstract and main paper), include the recommendation

Thank you for the remark. We include the recommendation both in the abstract and main paper.

-There are many grammar and typo issues that you need to address.

Thank you for the remark. We reviewed the manuscript and edited it by a person proficient in written English and we corrected any spelling and grammar errors that we identified.

- as pointed out by Reviewers 2 and 3, you need to revise your methods section

Thank you for the remark. We revised the method section as well as the entire manuscript.

- as pointed out by Reviewer 1, you need to rework on your discussion sections considering your results and the study area's context and current implementation strategies/policies, and other available evidence within or outside Ethiopia. This also applies to the recommendations.

Thank you for the remark. We rewritten in briefly and in detail the discussion part as indicated.

- Overall, you need to restructure your manuscript and follow the journal guideline. You can check some of the published papers for guidance.

Thank you for the remark. We had restructured the entire manuscript in detail now.

**Authors’ response for Reviewer #2:**

The text still needs some refinements for publication. In the summary, it is appropriate to include only the sample that was used, 448 women, and not the total that was eligible, 651 women. Finally, consult the Medical Subject Headings (MESH) of the keywords of the study. In the inclusion method, the simple random sampling technique was used to select controls and obtain the final sample. In the results, rewrite the initial paragraph, as the construction does not make it clear whether the results of the sociodemographic characteristics are related to 651 or 448 women in the study. In figure 1, the value informed in the text for the potentially eligible population is different, in the text 651 medical records and in figure 1- 569 medical records.

Thank you for the remark. We include only the study sample size (448 medical charts with APH) as indicated,. We also totally rewrote the keywords according to Medical Subject Headings. The information from Figure 1 and in the text had crossmatched.

**Authors’ response for Reviewer #3:**

The authors have conducted an interesting analysis about an important topic: DETERMINANTS OF FETO-MATERNAL OUTCOMES OF ANTEPARTUM HEMORRHAGE AMONG WOMEN WHO GAVE BIRTH IN AWI ZONE PUBLIC HOSPITALS, ETHIOPIA, 2021. I encourage them to consider the following points as they revise the manuscript.
**Title:** The title is clear and relevant.

**Abstract**
1. Background: Please add clear justification/gap why you conduct this study; there was other studies done in different areas of Ethiopia?

Thank you for the remark. We clarified our justification in clear and precise manner.

2. Conclusion: Please add what is your recommendation based on your findings

Thank you for the remark. Based on our study findings we had added the pertinent

recommendations.

**Introduction**
1. Please better to add what figure shows about late pregnancy bleeding/APH in Ethiopia.

Thank you for the remark.

2. Better to add clear gap… what makes your study differ from others/why you intended to conduct this study area.

Thank you for the remark. Based on our study findings we had added the relevant

recommendations.
**Methods**
1. Needs to be clarify ‘‘…..the final calculated sample size was 448 (224 cases and 244 controls)’’.

Thank you for the remark. We clarified how we determined the sample size.

2. Clearly rewrite the inclusion criteria….. ‘‘Inclusion Criteria Women diagnosed with APH and singleton delivery ≥weeks of gestation and who had full information needed for the study were included’’

Thank you for the remark. We have rewritten this sentence in a clarified way.

3. Why you excluded women who diagnosed with multiple pregnancy?

Thank you for the remark. The reason why we exclude multiple pregnancies is that it is difficult to identify the poor fetomaternal outcomes whether it is from Antepartum hemorrhage or multiple pregnancies. Because multiple pregnancies by itself also complicate fetomaternal outcomes.
4. Rewrite the operational definition of Antepartum hemorrhage it’s not correct

Thank you for the remark. We had defined the term correctly now.

**Result**
1. What does it mean ….651 were diagnosed and managed, and 2.6% of women get complications.

Thank you for the remark. This sentence had rewritten in a clear, precise, and correct way. We mean that there were 25,152 deliveries in the five years at Awi Zone public hospitals. Among these, 651 had antepartum hemorrhage which accounts for 2.6% of the total deliveries. After rejecting cards with incomplete variables, we had 569 complete eligible cards. From these eligible cards, 224 cases were completely ascertained and 224 controls were selected by random method.
2. Please rewrite again…. ‘‘Women residing in rural areas were 70.6% times more likely to develop poor feto-maternal outcomes compared to women residing in urban areas [AOR= 1.706, 95% CI 1.09, 2.66].’’

Thank you for the remark. This sentence had been rewritten correctly.

3. How ‘‘LNMP remember’’ could be a predictor of Feto-maternal outcomes regarding APH?

Thank you for the remark. The last normal menstrual period (LNMP) is an important parameter to calculate gestational age if it is reliable. In our study, it was a good indicator of fetal outcomes as a premature or mature fetus by gestational age using last normal menstrual period.

**Discussion**
1. Needs rewriting …. Poor justification.

Thank you for the remark. We had elaborate more regarding our findings in detail way now.
2. Please add the limitations of this study.

Thank you for the remark. We had added the limitations for this study.

**Conclusion:**
1. Please better to add and correlate your recommendation with the findings

Thank you for the remark. Now we had added recommendations directly correlated with our study findings.
