# Supplementary material for: Determinants of feto-maternal outcomes of antepartum hemorrhage among women who gave birth in Awi zone public hospitals, Ethiopia. A case-control study
Source: PLoS One. 2024 Jul 8;19(7):e0297700. doi: 10.1371/journal.pone.0297700 (PMC11230564; doi:10.1371/journal.pone.0297700)
Supplement: Attachment — Submitted filename: Response to Reviewers.docx [file pone.0297700.s006.docx]

**Author response**

**Manuscript ID:** PONE-D-22-15417R2

**Manuscript title:** Determinants of Feto-Maternal Outcomes of Antepartum Hemorrhage Among Women Who Gave Birth in Awi Zone Public Hospitals, Ethiopia, 2021.

**Dear Editor**

Thank you for giving us the opportunity to submit a revised draft of our manuscript “Determinants of Feto-Maternal Outcomes of Antepartum Hemorrhage Among Women Who Gave Birth in Awi Zone Public Hospitals, Ethiopia, 2021 to PLOS ONE. We appreciate the time and effort that you and the reviewers have dedicated to providing your fruitful feedback on our manuscript. We are grateful to the reviewers for their insightful comments on our manuscript. We have been able to incorporate changes to reflect most of the suggestions provided by the reviewers. We have highlighted (colored) the changes within the manuscript.

Here is a point-by-point response to the reviewers^’^ and academic editor’s comments.

Thank you!

Ambaye Minayehu (MSc)

On the behalf of all authors.

**reviewer (3)' Comments to Author:**

1. Needs to be clarify ‘‘the final calculated sample size was 448 (224 cases and 244 controls which yields 468)’’.

**Response:** This is an editing error, in fact the final calculated sample size was 448 medical record charts, in which (224 cases and 224 controls).

2. Why you excluded women who diagnosed with multiple pregnancy?

**Response:** We exclude multiple pregnancy b/c it is difficult to identify the outcomes wither it is by APH or multiple pregnancy. In fact, multiple pregnancy may end up with pre term labour, low birth weight, intrauterine growth retardation and the like which similar with APH outcomes. This is the main reason why we exclude multiple pregnancy in our study.

Academic Editor’s **Comment to Author:**

1. Abstract: the background information is unnecessarily long. Avoid definitions of APH and add take home messages grounded on this study.

**Response:** We are grateful for the valuable suggestions and feedback from academic editor. We agree with this and have incorporate your suggestion at abstract part and throughout the manuscript.

2. The Introduction section mixes the problem statement with significance and rationale of the study. I would suggest restricting as 1) problem statement (that is magnitude of APH, its consequences/outcomes, etc.); 2) what is known and not in the literature (including risk factors or determinants and national efforts). In this case, the last sentence of the first paragraph should come here; 3) and then the significance and/or rationale of the study could follow.

**Response:** We appreciate the constructive and insightful comments of academic editor’s. We agree with editor’s comment and have revised the manuscript accordingly.

3. Variables and analysis: How distance traveled is measured? Residence could interact with other variables like delay to seek care, ANC follow-up, etc. As such, during analysis, in addition to the collinearity check, I was expecting an examination of the effect modification or interaction effect.

**Response:** Thank you for the remark. The distance traveled is measured by kilometer as classified near (with in ten kilometer of health facility) and far (more than ten kilometer) from health facility. Our classification was based on other literature classification. We had checked collinearity effect by using the variance-inflation factor (VIF).

4. Limitations: Any limitations regarding the missingness of data or records? And what specific variables were not measured due to the retrospective nature of the study.

**Response:** We appreciate the constructive and insightful comments of academic editor’s. In our study there was a limitation due to the retrospective nature specifically, smoking which is risk factor for Abruptio placenta.

5. Discussion: Please summarize the key findings of this study in the first paragraph and then compare the findings with the literature and discuss their implications in subsequent paragraphs. For instance, paragraphs 2, 3, and 4 are not primary outcomes of this study. You might discuss them later. Discuss the key determinants first.

**Response:** We appreciate the constructive and insightful comments of academic editor’s. We agree with editor’s comment and have revised the manuscript accordingly.

6. Minor: I don’t think charts or records are source and study populations, rather mothers Please correct accordingly.

**Response:** Thank you for this suggestion. However, in our study, this would not be possible because our study was retrospective by using secondary data from mother’s medical records.

7. Regarding health system-related variables, I feel some of them are obstetric or clinical factors like ANC follow-up and prolonged hospital stay. Think of it.

**Response:** We appreciate the constructive and insightful comments of academic editor’s. They are obstetric or clinical factors but we put in health system-related variables based on related literature and service provided. For example, when the pregnant women stay more than expected date due to health system factor and in appropriate ANC service delivery in health facility after reaching health facility. On the behalf of this, thus variables can be grouped as health system related factors.

8. Some of the recommendations are not grounded in this study.

**Response:** we agree with this and have incorporate your suggestion at recommendation part.

Lastly we thank the reviewers for their constructive and helpful comments, which have significantly improved the quality and clarity of our manuscript and editors for handling our manuscript and facilitating the communication with reviewers.
